# War on the Hookworm

**Published:** 1912-08

**Authors:** 


### Abstracts and Selections.

### War on the Hookworm.

Concerning plans for the Statewide anti-hookworm campaign about to be launched, Dr. M. H. Boerner, the Austin physician who is to have charge of the work, said:

"The hookworm commission of the State Board of Health, which was organized to eradicate hookworm infection and disease from this State, is directing all of its efforts toward three tasks, namely: Determining the exact distribution and degree of infection, getting the sufferers treated, removing the cause of infection by putting a stop to soil pollution. In fact, all of the work is educational, even the treating of the people; every person treated will become the teacher of his neighbors; the final aim of all of our work is to help the people to stamp out hookworm infection by putting a stop to soil pollution.

"Ten other Southern States have been conducting a similar campaign in their respective States during the past two years, with the following results: Among the rural children of the school age the infection with hookworm has been definitely determined to range from 2½ to 90 2-10 per cent; all of these infected persons have received treatment, from either private physicians or from physicians of the hookworm commission, and they have been instructed how to prevent reinfection by improving sanitation.

"Hookworm disease is prevalent in many of the counties of East Texas, the percentage of infection ranging from 1 to 50 and over in many sections. The economic loss resulting from this disease is enormous, because a severe infection with hookworms will make invalids of the laborers, especially in the rural districts; hence the 'producers' become consumers. The disease also has a marked retarding effect on education and civilization, not only by keeping the children out of school, because of the physical disability, but also by its working subtly through long periods of time, producing accumulative results—physical, intellectual, economic and moral—which are handed down as an increasing handicap from generation to generation.

"The great undertaking of the State Board of Health should be heartily endorsed by every man and woman in Texas, and should receive united co-operation in every way to advance this public health movement to improve the sanitation in our rural schools and dwellings. Such moral support carries with it a weight which no appropriation of money from the outside could have, in stamping out hookworm infection which is such a serious menace to public welfare."—*S. B. Bulletin.*

